# *ErmF* and *ereD* Are Responsible for Erythromycin Resistance in *Riemerella anatipestifer*

**DOI:** 10.1371/journal.pone.0131078

**Published:** 2015-06-24

**Authors:** Linlin Xing, Hui Yu, Jingjing Qi, Pan Jiang, Bingqing Sun, Junsheng Cui, Changcan Ou, Weishan Chang, Qinghai Hu

**Affiliations:** 1 Shanghai Veterinary Research Institute, the Chinese Academy of Agricultural Sciences, 518 Ziyue Road, Shanghai, 200241, China; 2 College of Animal Science and Veterinary Medicine, Shandong Agricultural University, No. 61, Daizong Road, Tai’an, 271018, China; University of Minnesota, UNITED STATES

## Abstract

To investigate the genetic basis of erythromycin resistance in *Riemerella anatipestifer*, the MIC to erythromycin of 79 *R*. *anatipestifer* isolates from China and one typed strain, ATCC11845, were evaluated. The results showed that 43 of 80 (53.8%) of the tested *R*. *anatipestifer* strains showed resistance to erythromycin, and 30 of 43 erythromycin-resistant *R*. *anatipestifer* strains carried *ermF* or *ermFU* with an MIC in the range of 32–2048 μg/ml, while the other 13 strains carrying the *ereD* gene exhibited an MIC of 4–16 μg/ml. Of 30 *ermF* + *R*. *anatipestifer* strains, 27 (90.0%) carried the *ermFU* gene which may have been derived from the CTnDOT-like element, while three other strains carried *ermF* from transposon Tn4351. Moreover, sequence analysis revealed that *ermF*, *ermFU*, and *ereD* were located within the multiresistance region of the *R*. *anatipestifer* genome.

## Introduction

*Riemerella anatipestifer* is one of two species in the genus *Riemerella* within the family *Flavobacteriaceae*, which is one of the largest branches in the phylum *Bacteroidetes* [1, 2]. *R*. *anatipestifer* infection is a contagious disease of domestic ducks, geese, turkeys, and various other domestic and wild birds that poses a substantial threat to the duck industry worldwide and accounts for significant economic losses [3].

Erythromycin inhibits bacterial protein synthesis by binding at the exit tunnel of the 50S ribosomal subunit, resulting in the subsequent abortion of the growth of nascent peptide chains. Years ago, many *R*. *anatipestifer* clinical isolates were found to be sensitive to erythromycin [4], thus this antibiotic has been used to successfully treat *R*. *anatipestifer* infection in some duck flocks. However, erythromycin treatment failures have been noted on several occasions in the past few years. The growing increase in the rates of erythromycin-resistant *R*. *anatipestifer* isolates in recent years is alarming and the mechanism of erythromycin resistance in *R*. *anatipestifer* has not been described.

Three major mechanisms of erythromycin resistance have been identified in Gram-negative and-positive bacteria [5, 6]. The most well-known mechanism is the target-site modification of the 50S ribosomal subunit, which is mainly mediated by methylases encoded by the erythromycin ribosomal methylase *(erm)* gene and this methylation also causes resistance to lincosamides and streptogramin B antibiotics (MLS) [7]. The second described mechanism is the synthesis and activity of erythromycin inactivating enzymes, such as erythromycin esterase [8, 9]. The third known cause of erythromycin resistance is the active removal of the antibiotic by efflux systems, which maintains the intracellular antibiotic concentration at a subtoxic level that does not affect bacterial cell growth [5, 10].

So far, there have been relatively few studies on erythromycin resistance in bacteria in the family *Flavobacteriaceae*. However, in *Bacteroides*, which belong to the family *Bacteroidaceae* in the phylum *Bacteroidetes*, several *erm* genes (*ermB*, *ermF and ermG*) have been found [11]. In addition, our previous results demonstrated that the *ermF* gene may be expressed in *R*. *anatipestifer* and the conjugative transposon Tn4351 can be transferred to and randomly inserted into the genome of *R*. *anatipestifer* [12]. In this study, for the first time, our results showed that erythromycin resistance in *R*. *anatipestifer* was due to the presence of the *ermF*, *ermFU*, and *ereD* genes in the bacterial genome.

## Materials and Methods

### Bacterial strains and growth conditions

From 1996 to 2014, a total of 79 *R*. *anatipestifer* isolates were isolated from sick ducklings in China (S1 Table). *R*. *anatipestifer* type strain ATCC11845 and *Escherichia coli* strain ATCC25922 were obtained from the American Type Culture Collection (ATCC; Manassas, VA, USA). *R*. *anatipestifer* strains were cultured at 37°C in tryptic soybean broth (TSB; TSB, Difco Laboratories, Detroit, MI, USA) or agar in an atmosphere of 5% CO_2_. *E*. *coli* strains were grown in Luria-Bertani broth or agar at 37°C.

### Antibiotics susceptibility testing

Erythromycin susceptibility tests for each strain were performed in 96-well microtitre plates (Corning Incorporated, Corning, NY, USA) by determination of the minimum inhibitory concentration (MIC) value of erythromycin, as described previously [5]. *E*. *coli* ATCC 25922 isolates were used for quality control. Erythromycin was serially diluted two-fold in TSB broth to obtain antibiotic solutions with concentrations ranging between 4096 and 0.0625 μg/ml. The turbidity of the inoculum was adjusted to 10^7^ CFU/ml (100 μl/well). An inoculated broth containing no antibiotic was included as a growth control and a tube of uninoculated broth was used as a sterility control. The microplates were incubated at 37°C for 24 h. The lowest concentration of erythromycin that inhibited bacterial growth was considered as the MIC. Due to the lack of Clinical and Laboratory Standards Institute (CLSI)-approved erythromycin breakpoints applicable to *R*. *anatipestifer* and, moreover, in our previous study, 1 μg/ml of erythromycin was successfully used to select random Tn4351 transposon mutants of *R*. *anatipestifer* strain CH3[12], strains with an MIC of erythromycin of ≤0.25 μg/ml were considered susceptible, 0.5 μg/ml as intermediate, and ≥1 μg/ml as resistant as per the CLSI-approved criteria for *Streptococcus spp*. [13]. These criteria were further confirmed using the disc diffusion test with 15-μg erythromycin disks (Hangzhou Microbiological Co., Hangzhou, China). The final MIC value was estimated based on the average of at least three measurements.

### Detection of erythromycin resistance genes in *R*. *anatipestifer* isolates

Our previous results confirmed *ermF* expression in *R*. *anatipestifer* [12]. In addition, we found an erythromycin esterase gene in the genome of *R*. *anatipestifer* strain CH-2 (accession number: CP004020), which contained an erythromycin esterase domain (pfam05139, superfamily cl17110) and displayed 15.0%–25.5% amino acid identity to that of *ereA* (DQ157752, NC_015844), *ereB* (NC_008571, NC_017803), and *ereC* (NC_019153, NC_022657). We designated this erythromycin esterase gene as *ereD*.

To determine whether *ermF* or *ereD* was harboured in *R*. *anatipestifer*, DNA extracted from boiled *R*. *anatipestifer* bacteria was used as a DNA template for detection of the *ermF and ereD* genes by PCR. In addition, to further determine that the identified erythromycin resistance gene cassettes in *R*. *anatipestifer* were located within the genome or plasmid, the genomic DNA of erythromycin-resistant strains was isolated using the TIANamp Bacteria DNA Kit (Tiangen Biotech (Beijing) Co., Ltd., Beijing China) and the plasmids were extracted using the TIANprep Midi Plasmid Kit (Tiangen Biotech). The *ermF and ereD* genes were PCR-amplified using genomic DNA or plasmid as templates, respectively. In addition, to determine the type of *ermF* carried by different *ermF* ^+^ *R*. *anatipestifer* strains, the upstream sequence of *ermF* gene was PCR-amplified using primers ermFU P1 plus ermFU P2 for *ermFU*, and ermF P1' plus ermFU for *ermF*, respectively. DNA sequencing was used to identify the PCR products.

In addition, to determine whether *R*. *anatipestifer* carried other genes that convey erythromycin resistance in other bacterial species, PCR was used to test for the presence of the *ermA*, *ermB*, *ermC*, *ermD*, *ermE*, *ermG*, *ermT*, *ermX*, *mphA*, *mphR*, and *msrA* genes in all *R*. *anatipestifer* isolates. The primers used in this study are listed in S2 Table.

### Genomic walking of the full *ermF* cassette of strain HXb2 and its flanking sequences

The open reading frames (ORFs) of the *ermF* gene were PCR-amplified from different *R*. *anatipestifer* strains and sequenced. Genome walking was performed to clone the upstream flanking sequence from strains with different erythromycin resistance levels according to the manufacturer’s instructions (Takara Biotech Co., Ltd, Dalian, China). In addition, differences in the upstream sequence of *ermF* types (*ermF*, *ermFU*, or *ermFS*) carried by *ermF* ^+^ *R*. *anatipestifer* strains were determined by PCR. Sequence analysis was performed using Lasergene 7.0 software (DNASTAR Inc., Madison, WI, USA). To determine how the *ermF* gene was transferred into the genome of *R*. *anatipestifer*, the genetic environment of the *ermFU* cassette within the genome of HXb2, which is the most virulent isolate identified in ducklings so far, was sequenced and analysed using the BLAST program (http://blast.ncbi.nlm.nih.gov/Blast.cgi).

### Transfer experiment

The *ermFU* cassette from *R*. *anatipestifer* strain HXb2, YXb15 and NJ4, the *ermF* cassette from strain YZ-1, and the *ereD* cassette from strain SX were amplified and cloned into the *E*. *coli*–*R*. *anatipestifer* shuttle vector pRES0 [14], respectively. Then, the recombinant plasmids were introduced by conjugation as previously described [15], respectively, into an erythromycin-susceptible *R*. *anatipestifer* strain CH3 to generate the recombinant strains CH3 (pRES-HXb2-ermFU), CH3 (pRES-YXb15-ermFU), CH3 (pRES-NJ4-ermFU), CH3 (pRES-YZ1-ermF), and CH3 (pRES-SX-ereD). The MICs of erythromycin and clindamycin for the wild-type and recombinant strains were measured as described above.

### Real-time PCR

To evaluate the expression of resistance genes at the transcriptional level in the wild-type and transferred strains, the bacteria were grown to log phase (OD_600nm_ ≈ 0.8–1.0), and total RNA was extracted using the RNeasy Mini kit (Qiagen, Hilden, Germany), followed by first-strand cDNA synthesis using the Sensiscript RT kit (Qiagen), according to the manufacturer's instructions. Quantitative real-time PCR was performed to measure the mRNA levels of *ermF*, *ermFU*, and *ereD* using SYBR green PCR master mix (Applied Biosystems, Foster City, CA, USA) and the primers listed in S2 Table. Relative quantification of gene expression was calculated using the ΔCT method based on the signal intensity of the PCR products according to the following formula: 2^-ΔCT^ = 2^-(sample Ct—normalizer Ct)^ (Ct = threshold cycle of real-time PCR). *TbdR1* (Riean_1024) was used as an endogenous control for sample normalization. Results are presented as fold-change relative to mRNA expression levels of the wild type strains.

### Nucleotide sequence accession numbers

The sequences of the *ermF* region of different *R*. *anatipestifer* strains, the *ereD* region of *R*. *anatipestifer* strain SX, and the multiresistance region (MRR) of strain HXb2, were deposited in the GenBank database under the accession numbers KP265714–KP265722 and KR857248-KR857269, respectively.

## Results and Discussion

### Erythromycin susceptibility testing

**Fig 1:**
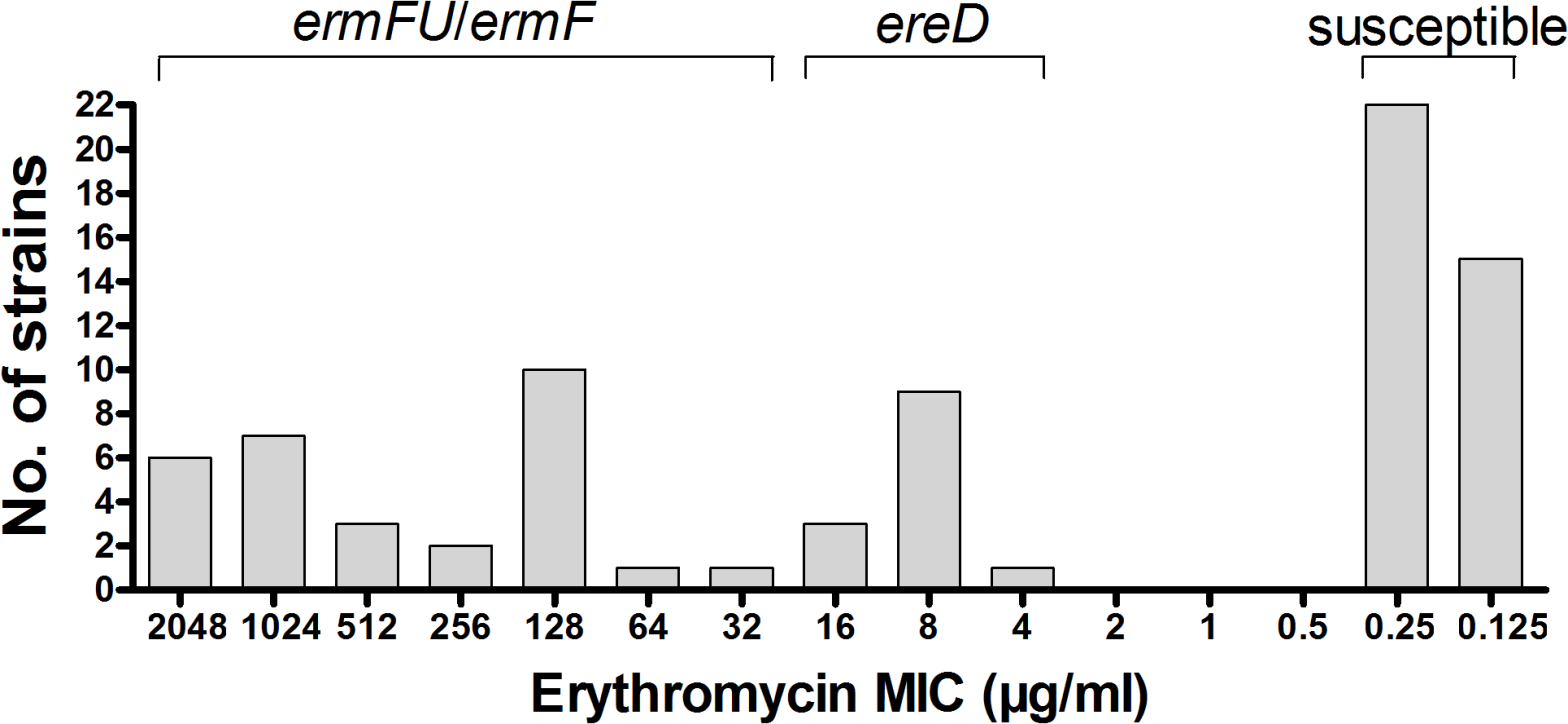
Distribution of *R*. *anatipestifer* strains with different MICs for erythromycin.

The erythromycin MICs of 80 *R*. *anatipestifer* strains ranged from 0.125 to 2048 μg/ml. As shown in Fig 1, 43 (53.8%) of the 80 tested strains exhibited resistance to erythromycin, while 37 (46.2%) were susceptible. Furthermore, 2 (8.3%) of 24 *R*. *anatipestifer* strains, which were isolated from sick ducklings in China between 1996 and 2004, were resistant to erythromycin, while 41 (77.4%) of 56 *R*. *anatipestifer* isolates obtained from 2005 to 2014 exhibited erythromycin resistance. These findings suggested that the percentage of isolates showing resistance to erythromycin greatly increased over the last 10 years. The increased use of erythromycin and other macrolides may have increased the selective pressure on bacterial populations [16], although the percentage of erythromycin-resistant *R*. *anatipestifer* isolates may vary among different duck farms in different regions in China.

### Prevalence of the *ermF* and *ereD* genes in *R*. *anatipestifer* strains

To evaluate the prevalence of the *ermF and ereD* genes in *R*. *anatipestifer* strains, the presence of these two genes in 80 *R*. *anatipestifer* strains were determined by PCR. The results showed that 30 (70.0%) of 43 erythromycin-resistant strains encoded the *ermF* gene. As shown in Fig 1, the range of MICs of 30 *ermF* ^+^ erythromycin-resistant strains were widely distributed from 32 to 2048 μg/ml, and 43.3% (13/30) of *ermF* ^+^ strains exhibited very high resistance (MIC ≥ 1024 μg/ml). On the other hand, all 13 *ermF* ^-^ erythromycin-resistant strains encoded the *ereD* gene, which exhibited relatively lower resistance with an MIC in the range of 4–16 μg/ml. Our results showed that both *ermF* and *ereD* were encoded by the *R*. *anatipestifer* genome, while none of the erythromycin-susceptible strains encoded the *ermF and ereD* genes. Moreover, none of the 80 tested *R*. *anatipestifer* strains harboured *mphA*, *mphR*, *msrA*, or other *erm* genes. These results suggested that *ermF* and *ereD* may be involved in erythromycin resistance in *R*. *anatipestifer* strains and the *ermF* gene, which codes for ribosomal methylase, was the most frequently encoded gene that determines erythromycin resistance in *R*. *anatipestifer*.

### Sequence analysis of the *ermF* gene in *R*. *anatipestifer*

**Fig 2:**
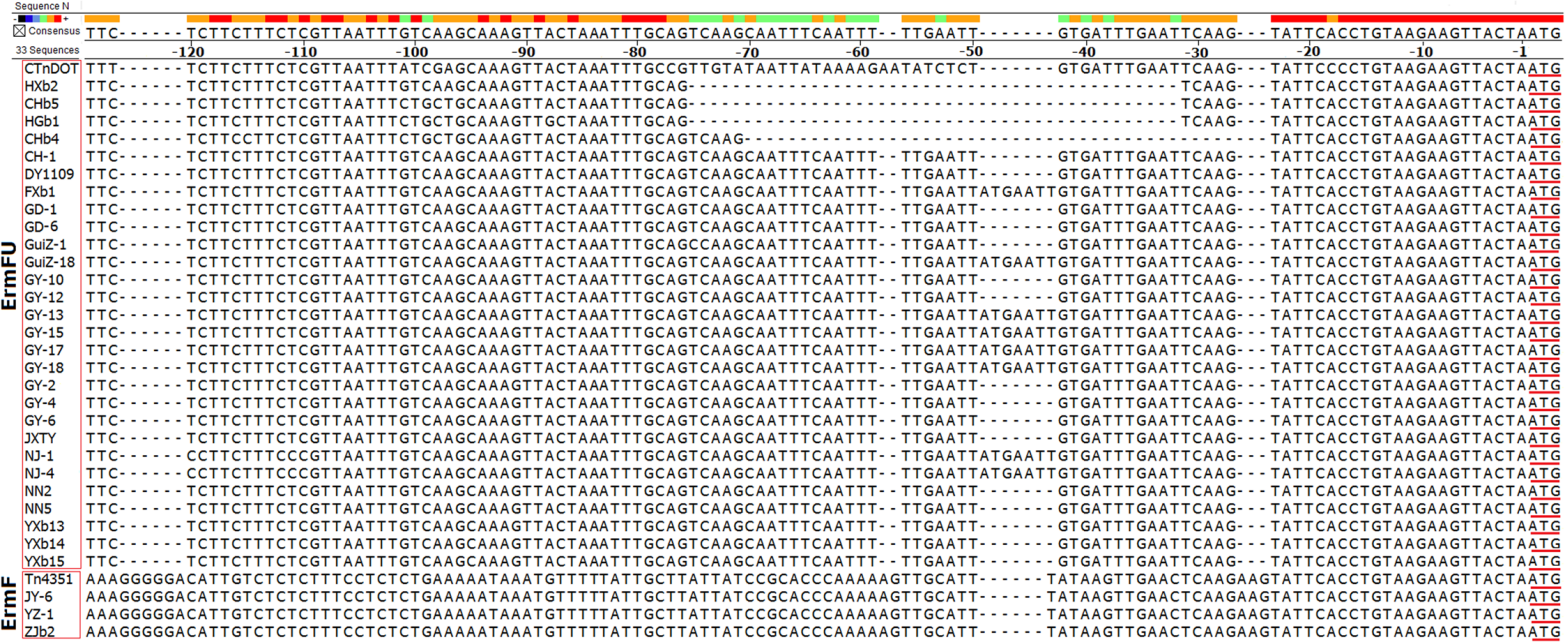
Clustal W alignment of the upstream sequences from the *ermF* / *ermFU* genes from CTnDOT, Tn4351, and different *R*. *anatipestifer* strains. The start codon ATG of *ermFU*/*ermF* gene was underlined.

Sequence analysis of the *ermF* ORFs revealed that the *ermF* gene from *ermF* ^*+*^ *R*. *anatipestifer* strains, which exhibit different erythromycin resistance levels, shared an amino acid homology of 98.5%–100%. There have been three *ermF* genes sequenced from the *Bacteroide frangilis* group: *ermF* (GenBank accession number: M14730), which is encoded by transposons Tn4351 and Tn4400 [17], *ermFS* (M17808), which is encoded by transposon Tn4551 [18], and *ermFU* (M62487, AJ311171), which is encoded by transposon Tn5030 and the conjugative transposon CTnDOT [19, 20]. In this study, according to differences within the upstream sequences, the *ermF* genes from different *R*. *anatipestifer* strains could be classified into two types: *ermF* and *ermFU*. As shown in Fig 2, 27 out of 30 (90.0%) *ermF* ^+^ *R*. *anatipestifer* strains tested in this study carried the *ermFU* gene, while only three strains carried *ermF*, which indicated that the *ermFU* gene was more widespread in *R*. *anatipestifer* than *ermF*, and the CTnDOT-like element (including Tn5030) significantly contributed to the dissemination of erythromycin resistance determinants in *R*. *anatipestifer*. Conjugal transfer is mostly responsible for the spread of resistance genes within the *Bacteroides* group [21]. Two types of conjugative elements have been identified in *Bacteroides*: plasmids and chromosomal elements. Transposons Tn4351, Tn4400, and Tn4551, which carry the e*rmF* or *ermFS* gene, were part of *Bacteroides* plasmids pBF4, pBFTM10, and pBI136, respectively [21], while Tn5030 or CTnDOT, which carry *ermFU*, were normally integrated into the bacterial chromosome [19, 20]. However, it was odd that all the erythromycin resistance genes identified in *R*. *anatipestifer* strains (*ermF*, *ermFU*, and *ereD* genes) were only found within the genomes. This may be the result of the evolution of *R*. *anatipestifer* because *Bacteroides* plasmids, such as pBF4, pBFTM10, and pBI136, may not be stably maintained in *R*. *anatipestifer* strains. In addition, CTnDOT contains essential mobilization genes (*mobA* and *mobB*) and other transfer (*tra*) genes [19], while Tn4351 does not. Therefore, CTnDOT-like elements may spread more easily in bacteria than Tn4351-like elements.

Sequence homology analysis showed that the upstream sequence of the *ermFU* gene from *R*. *anatipestifer* strains (from -1 to -220∼-262) shared 85.8%–88.7% homology with that of CTnDOT (from -1 to -256), and we found that the region at -29 to -70 of the upstream sequence of *ermFU* contained a 37-nt deletion (strains HXb2 and HGb1), a 2-nt deletion (strains GuiZ-1, YXb15, CH-1), or a 5-nt insertion (strain NJ-4), as compared with that in CTnDOT (Fig 2). In addition, there was a 25-nt replacement upstream of *ermFU* in different *R*. *anatipestifer* strains, as compared with that in CTnDOT. The -1 to -220∼-262 region upstream from the *ermFU* gene in *R*. *anatipestifer* strains may play roles in transcriptional control as a promoter. The above-mentioned sequence differences suggested that expression of the invading *ermFU* gene in *R*. *anatipestifer* may be dependent on certain sequence rearrangements within the transcription and/or translation start signals to accommodate *ermFU* gene expression in this host [20].

Conversely, as compared with the upstream sequence of *ermF* from transposon Tn4351, differences of only 1, 5, and 12 nt were found in strains YZ-1 (-1 to -590), ZJb2 (-1 to -677), and JY-6 (-1 to -618), respectively. Moreover, 564 bp (-27 to -590), 651 bp (-27 to -677), and 592 bp (-27 to -618) of the upstream sequences of the *ermF* gene from strains YZ-1, ZJb2, and JY-6 were the truncated forms of the IS4351R sequence of transposon Tn4351. Although the existing part of an IS4351 element may also be because the transcriptional start site for the *ermF* gene was contained within this insertion sequence (IS) element [17], our findings suggested that the *ermF* regions of these *R*. *anatipestifer* strains were derived from Tn4351. Both the *Bacteroides* and *Riemerella* genera belong to the phylum *Bacteroidetes*, which compose the most substantial portion of animal and human gastrointestinal flora. Therefore, for *R*. *anatipestifer*, *Bacteroides* could serve as reservoirs of antibiotic resistance genes and other genes, while possibly passing them on to *R*. *anatipestifer*.

### The genetic environment of the *ermFU* gene in HXb2

**Fig 3:**
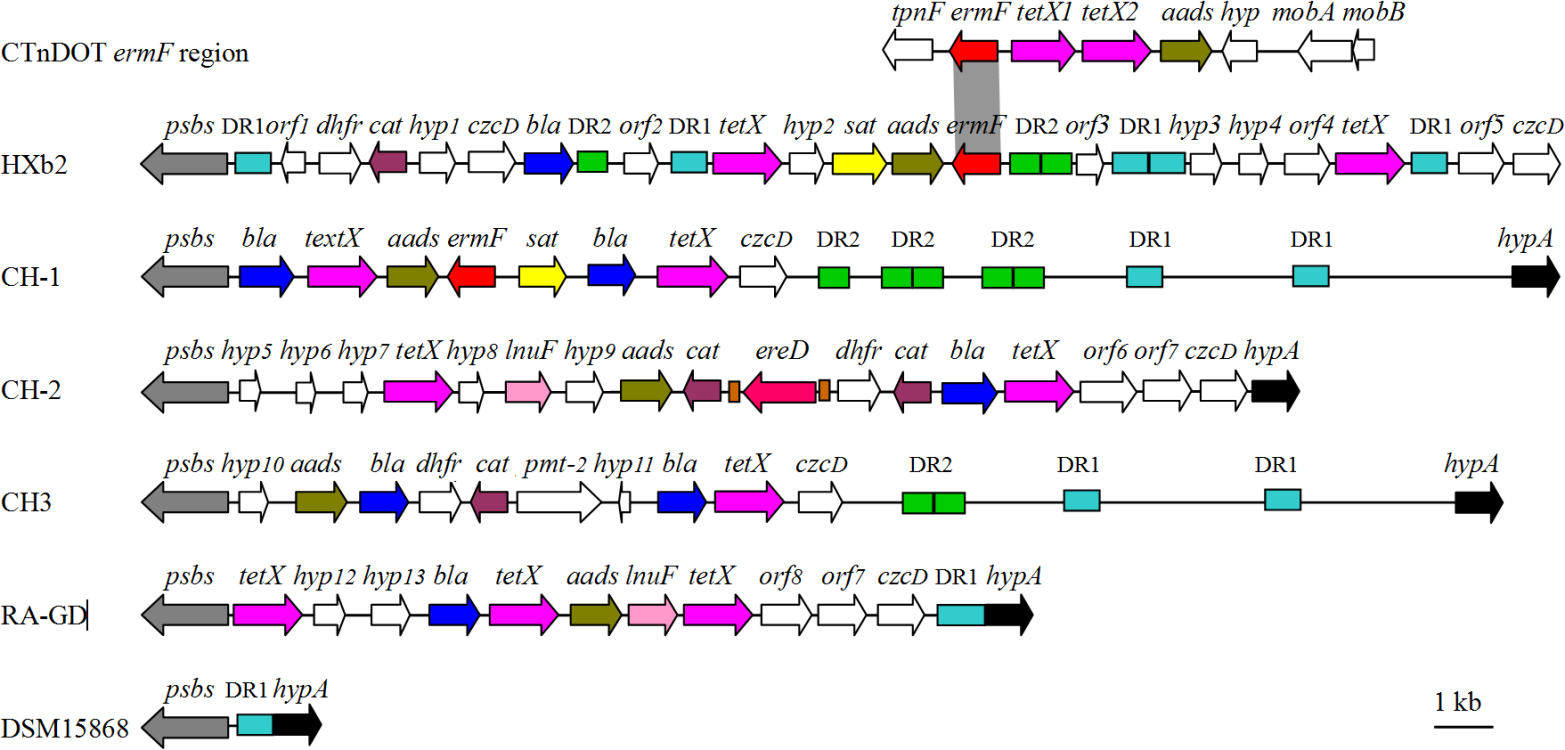
Genetic environment of the *ermFU* gene in HXb2 and structural comparison with the corresponding regions in CH-1, CH-2, CH3, RA-GD, and DSM15868. The arrows represent the positions and orientations of the genes. The boxes represent direct repeats (DR1-DR3), and the two boxes flanking *ereD* in the MRR of CH-2 represent DR3. *Orfs* 1–8 represent open reading frames with either unknown or unconfirmed functions. *Orfs* indicated as *hyp* may encode hypothetical proteins.

To gain insight into the genetic environment in HXb2, the *ermFU* gene and the flanking sequences were sequenced and compared with the corresponding regions in *R*. *anatipestifer* strains CH-1 (accession number: CP003787), CH-2 (CP004020), CH3 (CP006649), RA-GD (CP002562), and DSM15868 (CP002346). As shown in Fig 3, except for the type strain DSM15868, there was an 8–29-kb MRR in *R*. *anatipestifer* strains, and the *ermFU* gene of strains HXb2 and CH-1 were located in the MRR. Analysis of the MRR in *R*. *anatipestifer* strains HXb2, CH-1, CH-2, CH3, and RA-GD, and the corresponding region in strain DSM15868, indicated that the MRR in these strains was inserted into the non-coding region between the gene (*psbs*, Riean_1797) coding for polysaccharide biosynthesis protein and a gene (*hypA*, Riean_1798) coding for hypothetical protein. The insertion mechanism of the resistance genes at this specific region on the *R*. *anatipestifer* chromosome remains unknown, and only a few short DNA fragments (<45 bp) in the MRR of various other strains were found to share a high homology with certain ISs by online IS Finder software (https://www-is.biotoul.fr//), but we found a 601-bp (1988576–1989176) non-coding direct repeat (designated DR1) located at the intergenic region between *psbs* and *hypA* in DSM15868, and a total five copies of the DR1 were found scattered within a 30-kb region within this genome. DR1 was also found within corresponding regions of strains CH-1(2 copies), CH-2 (5 copies), CH3 (2 copies), RA-DA (5 copies), and HXb2 (5 copies in the MRR). Therefore, we speculated that the non-coding intergenic sequence between these two genes may be a recombinant hot spot for the insertion of antibiotic resistance genes. In addition, we found a non-coding tandem direct repeat (a 517-bp repeat unit designated DR2) located upstream of *ermFU* in the MRR of strain HXb2, and one copy of the DR2 repeat unit was also found between *bla* and *orf2* in this MRR (Fig 3). Moreover, tandem DR2 repeats were also found in the MRR of strains CH-1 (two copies) and CH3 (one copy), and 1–4 copies of the DR2 repeat unit were found in the MRR or other regions within the genomes of other *R*. *anatipestifer* strains.

In addition, the *aads-ermFU* region in the MRR in strain HXb2 shared an identity of 99.4% to that of strain CH-1, and the 199-bp junction sequences between the two genes were almost identical, with only a 1-nt replacement found, while nt sequences at the right and left junctions of the *aads-ermFU* region were obviously different. These findings suggest that the *aads and ermFU* genes may be co-transferred into *R*. *anatipestifer*. However, the *aads-ermFU* region was not found in other plasmids, CTns or bacterial genomes, while both the *aads and ermFU* genes were present in CTnDOT and shared 97%–99% identity to those in HXb2 and CH-1, while in CTnDOT, they were separated by genes *tetX1* and *tetX2*, and their orientations were also different from those in *R*. *anatipestifer* strains HXb2 and CH-1. These findings suggest that perhaps the *aads-ermFU* region in *R*. *anatipestifer* was derived from a new CTnDOT-like element which has not yet been discovered.

Sequence analysis showed that the *ereD* determinant in the CH-2 genome was flanked by the 217-bp non-coding direct repeat sequence DR3. In *R*. *anatipestifer* plasmids pRA0511 and pRA0846, we also identified the DR3 sequence, which was located at the left and right flanking regions of the *text-cat* genes and *floR*-truncated *sat* genes, respectively [22, 23]. The appearance of DR1–DR3 in the MRR indicated that these direct repeats may play an important role in transferring resistant genes or others into the genome of *R*. *anatipestifer*. In addition, the antibiotic resistance gene order and copy number in the MRR from different *R*. *anatipestifer* strains also differed, which suggested that these antibiotic resistance genes may be introduced into the MRR though several recombination events.

### Transfer of erythromycin resistance genes

To further determine whether the identified *ermF*, *ermFU*, and *ereD* genes were responsible for erythromycin resistance in *R*. *anatipestifer*, five recombinant shuttle plasmids carrying the *ermF*, *ermFU*, and *ereD* cassettes were introduced into an erythromycin-susceptible *R*. *anatipestifer* CH3. The results of MICs showed that all five recombinant strains, CH3 (pRES-HXb2-ermFU), CH3 (pRES-YXb15-ermFU), CH3 (pRES-NJ4-ermFU), CH3 (pRES-YZ-1-ermF), and CH3 (pRES-SX-ereD), exhibited erythromycin resistance, and four *ermF* carrying strains, CH3 (pRES-HXb2-ermFU), CH3 (pRES-YXb15-ermFU), CH3 (pRES-NJ4-ermFU), and CH3 (pRES-YZ-1-ermF), showed high resistance to clindamycin, while the CH3 carrying plasmid pRES-SX-ereD exhibited no resistance to clindamycin (Table 1). These findings suggested, just as with other bacteria, that *ermF* and *ermFU* in *R*. *anatipestifer* conferred resistance to the macrolide-lincosamide-streptogramin B group of antibiotics [17]. It is interesting that strain YZ-1 exhibited low erythromycin resistance with an MIC of 32 μg/ml, while CH3 (pRES-YZ-1-ermF), which carries the *ermF* gene cassette from strain YZ-1, exhibited high resistance to erythromycin with an MIC of 2048 μg/ml. Moreover, the mRNA level of *ermF* increased by 84.38 ± 37.21-fold in the transferred strain CH3 (pRES-YZ-1-ermF), as compared to that in the wild-type strain YZ-1 (S1 Fig). On the other hand, on behalf of the different changes (nucleotide acid deletion, insertion or replacement) at the upstream region of *ermFU* gene in *R*. *anatipestifer* strains, the *ermFU* cassettes of YXb15, HXb2 and NJ4 were transferred into CH3 respectively. Although YXb15 has the highest level of resistance to erythromycin among YXb15, HXb2 and NJ4, the mRNA expression of *ermFU* in HXb2 and CH3 (pRES-HXb2-ermFU) at the log phase (OD_600nm_ ≈ 0.8–1.0) was significantly higher than that in strains YXb15, CH3 (pRES-YXb15-ermFU), NJ4, and CH3 (pRES-NJ4-ermFU) (S2 and S3 Figs). Therefore, multiple mechanisms may be involved in the expression of the *ermFU* determinants in *R*. *anatipestifer* strains, including regulation at the transcription, posttranscription or translation level, as found in other bacteria [24].

**Table 1:** Comparison of MICs for wild-type CH3, CH3 (pRES-HXb2-ermFU), CH3 (pRES-YZ-1-ermF), and CH3 (pRES-SX-ereD).

Genotype	Strain	MIC (μg/ml)	
		erythromycin	clindamycin
	CH3	0.125	0.0625
*ermFU*	HXb2	512	2048
	CH3 (pRES-HXb2-ermFU)	1024	>2048
	YXb15	2048	>2048
	CH3 (pRES-YXb15-ermFU)	2048	>2048
	NJ4	64	2048
	CH3 (pRES-NJ4-ermFU)	128	>2048
*ermF*	YZ-1	32	>2048
	CH3(pRES-YZ-1-ermF)	2048	2048
*ereD*	SX	16	256
	CH3(pRES-SX-ereD)	32	0.125

## Supporting Information

S1 Fig Real-time PCR for *ermF* and *ermFU* for mRNA expression in the transferred and wild-type strains. Relative *ermF* mRNA levels in strains YZ-1 and CH3(pRES-YZ-1-ermF).
(TIF)

S2 Fig Relative *ermFU* mRNA levels in the wild-type strains HXb2, YXb15 and NJ4, and the transferred strains CH3(pRES-HXb2-ermFU), CH3(pRES-YXb15-ermFU) and CH3(pRES-NJ4-ermFU) when the bacteria were grown in TSB at 37°C with shaking to absorbance 600 nm at 0.8. (TIF)

S3 Fig Relative *ermFU* mRNA levels when the bacteria were grown in TSB at 37°C with shaking to absorbance 600 nm at 1.0. (TIF)

S1 Table *Riemerella anatipestifer* strains used in this study. (DOC)

S2 Table Primers used in this study. (DOC)
