# Supplementary figures and images for: ErmF and ereD Are Responsible for Erythromycin Resistance in Riemerella anatipestifer
Source: PLoS One. 2015 Jun 24;10(6):e0131078. doi: 10.1371/journal.pone.0131078 (PMC4481100; doi:10.1371/journal.pone.0131078)

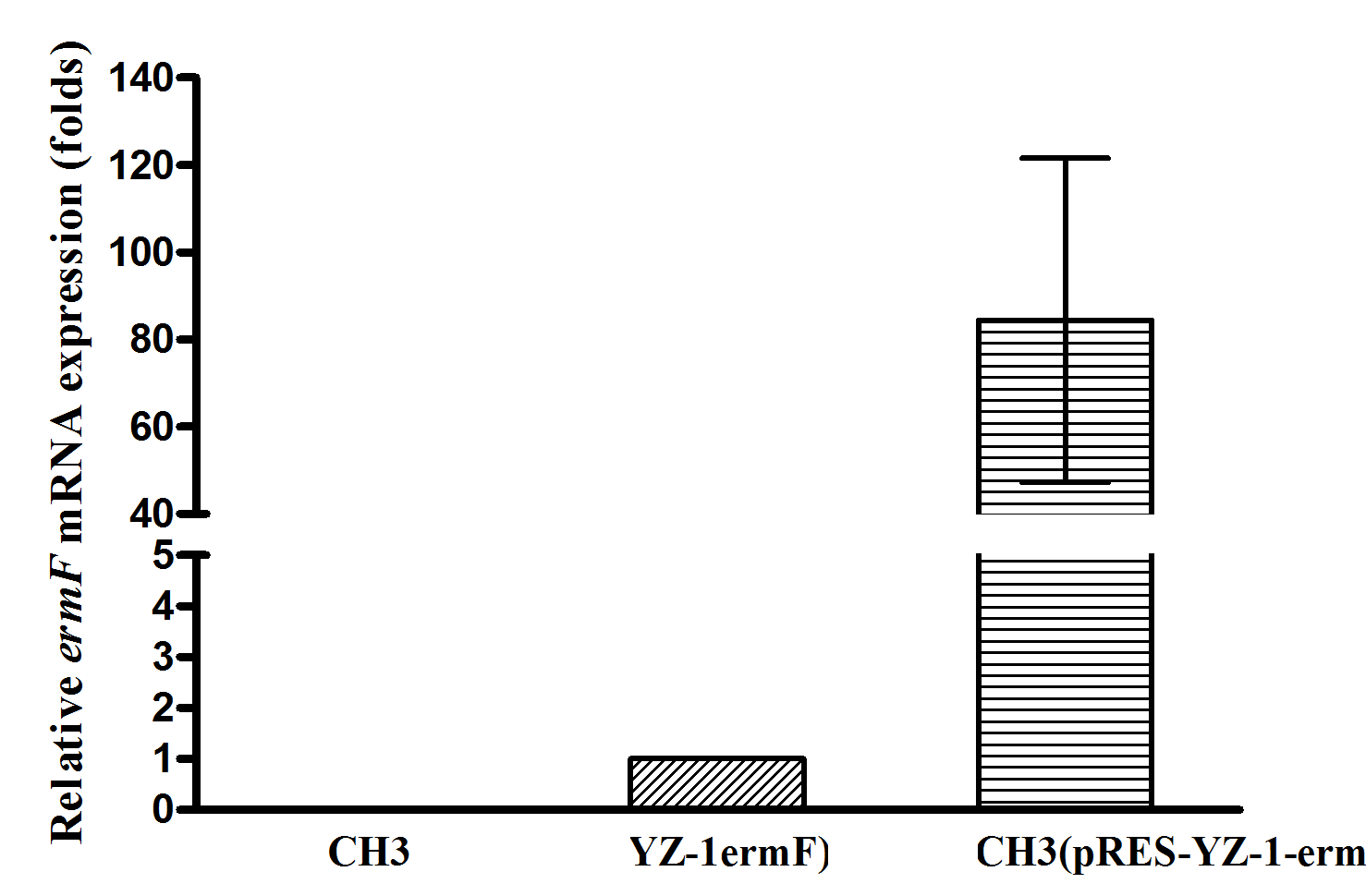

Supplement: S1 Fig — Relative ermF mRNA levels in strains YZ-1 and CH3(pRES-YZ-1-ermF). (TIF) [file pone.0131078.s001.tif]

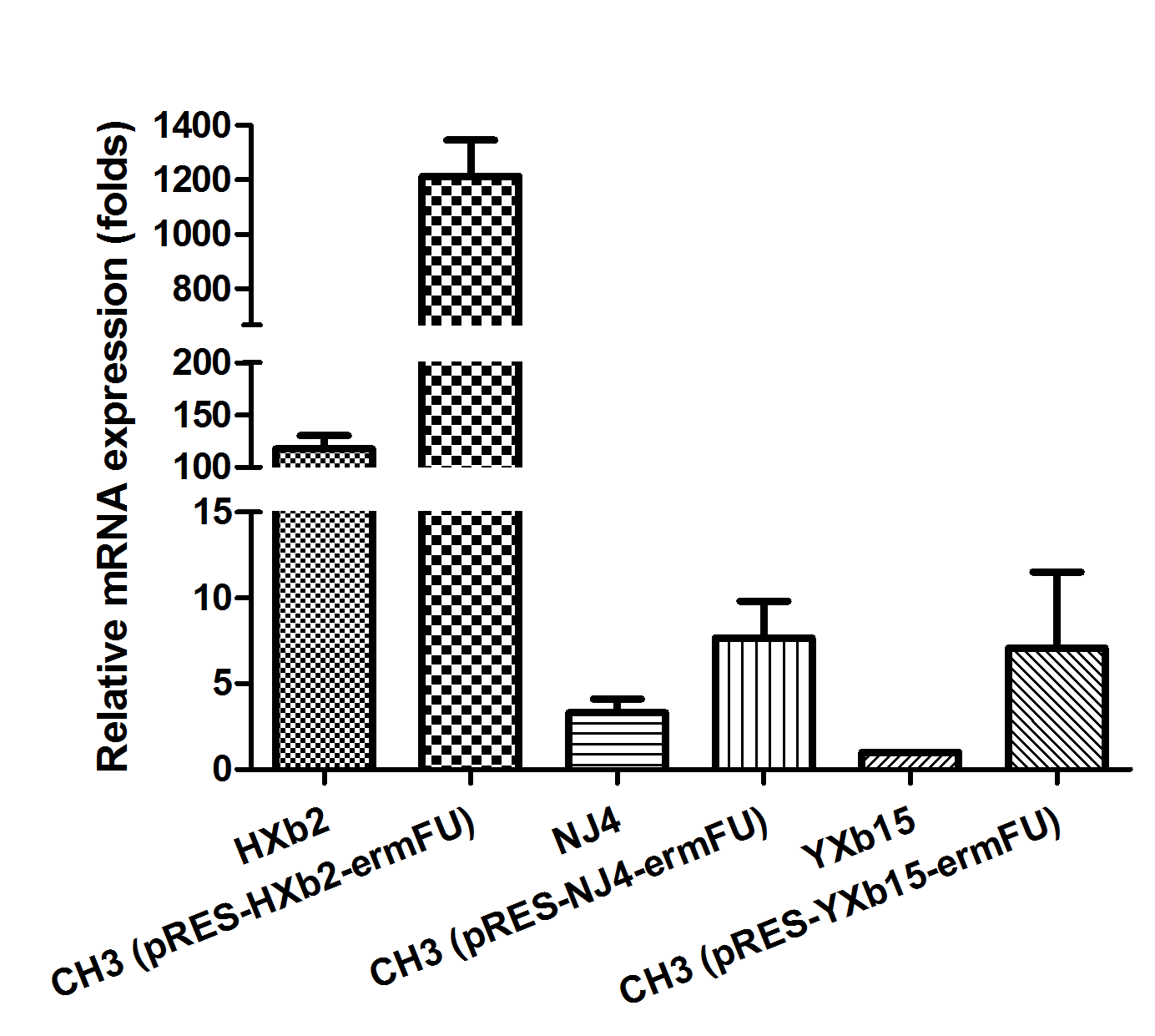

Supplement: S2 Fig — (TIF) [file pone.0131078.s002.tif]

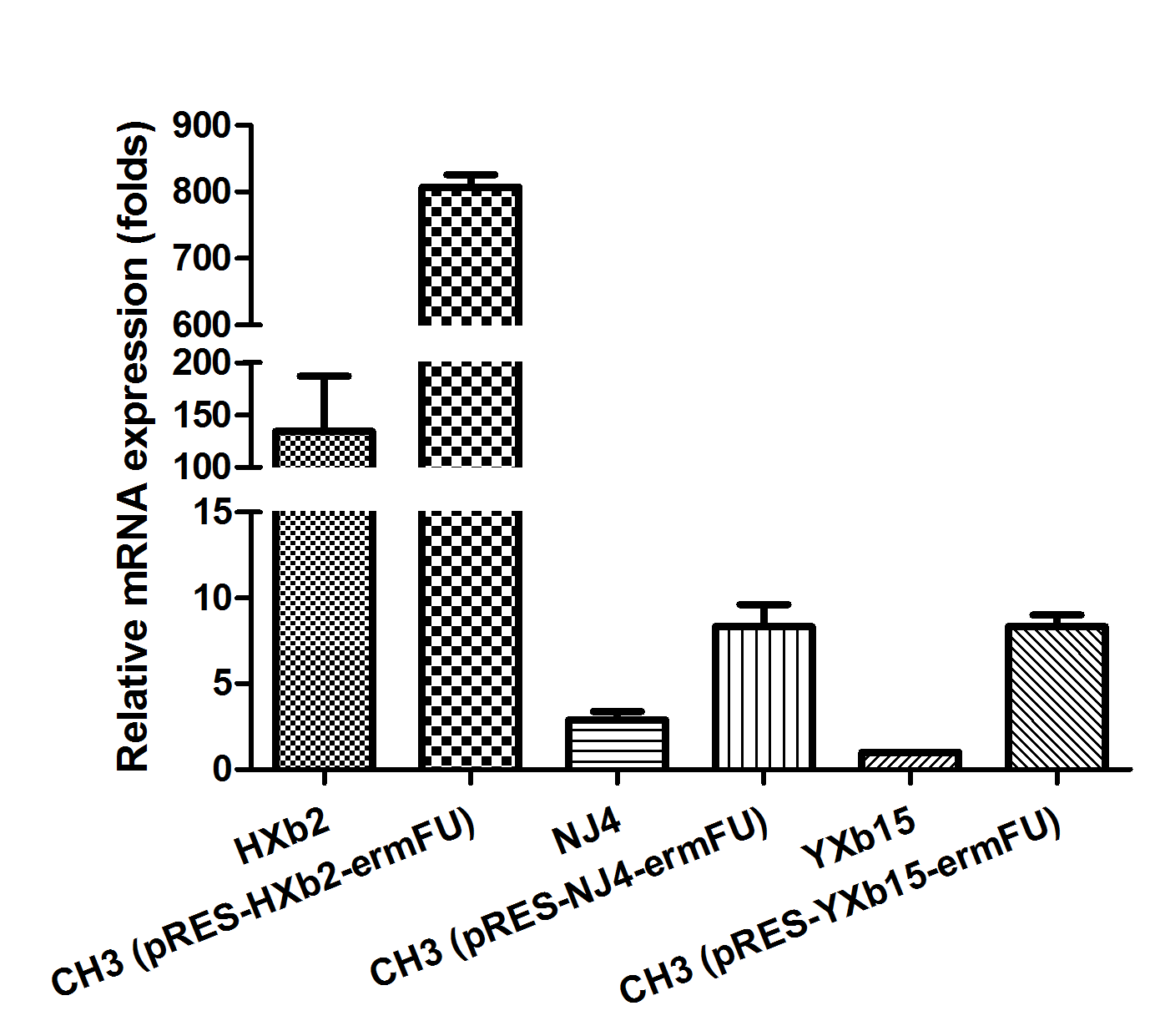

Supplement: S3 Fig — (TIF) [file pone.0131078.s003.tif]
