# Supplementary material for: ErmF and ereD Are Responsible for Erythromycin Resistance in Riemerella anatipestifer
Source: PLoS One. 2015 Jun 24;10(6):e0131078. doi: 10.1371/journal.pone.0131078 (PMC4481100; doi:10.1371/journal.pone.0131078)
Supplement: S1 Table — (DOC) [file pone.0131078.s004.doc]

**Table S1. *R. anatipestifer*** strains used in this study

| **NO.** | **Strains** | **Serotype** | **Year of isolation** | **MIC value of erythromycin** | ***ermF* or *ereD* type** |
| --- | --- | --- | --- | --- | --- |
| 1 | ATCC11845 | ND | - (type strain) | 0.25 µg/mL | - |
| 2 | GuiZ-1 | ND | 2012 | 2048 µg/mL | *ermFU* |
| 3 | YXb13 | 1 | 2007 | 2048 µg/mL | *ermFU* |
| 4 | YXb14 | 1 | 2007 | 2048 µg/mL | *ermFU* |
| 5 | DY-1109 | 2 | 2013 | 2048 µg/mL | *ermFU* |
| 6 | YXb15 | ND | 2009 | 2048 µg/mL | *ermFU* |
| 7 | ZJb2 | 1 | 2014 | 2048 µg/mL | *ermF* |
| 8 | JXTY | 1 | 2013 | 1024 µg/mL | *ermFU* |
| 9 | JY-6 | ND | 2008 | 1024 µg/mL | *ermF* |
| 10 | GD-6 | ND | 2008 | 1024 µg/mL | *ermFU* |
| 11 | GY-2 | ND | 2009 | 1024 µg/mL | *ermFU* |
| 12 | GY-4 | ND | 2010 | 1024 µg/mL | *ermFU* |
| 13 | GY-6 | ND | 2010 | 1024 µg/mL | *ermFU* |
| 14 | GY-10 | ND | 2012 | 1024 µg/mL | *ermFU* |
| 15 | HXb2 | 10 | 2000 | 512 µg/mL | *ermFU* |
| 16 | NN-2 | 1 | 2008 | 512 µg/mL | *ermFU* |
| 17 | GY-12 | ND | 2012 | 512 µg/mL | *ermFU* |
| 18 | GD-1 | ND | 2008 | 256 µg/mL | *ermFU* |
| 19 | GY-18 | 2 | 2012 | 256 µg/mL | *ermFU* |
| 20 | NJ-1 | 1 | 2008 | 128 µg/mL | *ermFU* |
| 21 | NN-5 | 2 | 2008 | 128 µg/mL | *ermFU* |
| 22 | GuiZ-18 | 2 | 2013 | 128 µg/mL | *ermFU* |
| 23 | FXb1 | ND | 2009 | 128 µg/mL | *ermFU* |
| 24 | HGb1 | ND | 2010 | 128 µg/mL | *ermFU* |
| 25 | CHb4 | ND | 2010 | 128 µg/mL | *ermFU* |
| 26 | CHb5 | ND | 2010 | 128 µg/mL | *ermFU* |
| 27 | GY-13 | 2 | 2012 | 128 µg/mL | *ermFU* |
| 28 | GY-15 | 2 | 2012 | 128 µg/mL | *ermFU* |
| 29 | GY-17 | 2 | 2012 | 128 µg/mL | *ermFU* |
| 30 | NJ-4 | 1 | 2008 | 64 µg/mL | *ermFU* |
| 31 | YZ-1 | 6 | 2008 | 32 µg/mL | *ermF* |
| 32 | SX | ND | 2010 | 16 µg/mL | *ereD* |
| 33 | JY-1 | 2 | 2008 | 16 µg/mL | *ereD* |
| 34 | GD-5 | 2 | 2008 | 16 µg/mL | *ereD* |
| 35 | XDb1 | ND | 2008 | 8 µg/mL | *ereD* |
| 36 | JY-5 | 2 | 2008 | 8 µg/mL | *ereD* |
| 37 | GD-3 | 2 | 2008 | 8 µg/mL | *ereD* |
| 38 | GD-7 | 2 | 2008 | 8 µg/mL | *ereD* |
| 39 | WWb2 | 2 | 2009 | 8 µg/mL | *ereD* |
| 40 | WWb3 | 2 | 2009 | 8 µg/mL | *ereD* |
| 41 | CFb1 | 2 | 2009 | 8 µg/mL | *ereD* |
| 42 | YZ-4 | 12 | 2008 | 8 µg/mL | *ereD* |
| 43 | YZb1 | 15 | 2002 | 8 µg/mL | *ereD* |
| 44 | YZ-3 | 8 | 2008 | 4 µg/mL | *ereD* |
| 45 | CH1 | 1 | 2001 | 0.25 µg/mL | - |
| 46 | YL4 | 1 | 2001 | 0.25 µg/mL | - |
| 47 | YXb12 | 1 | 2007 | 0.25 µg/mL | - |
| 48 | JY-4 | 1 | 2006 | 0.25 µg/mL | - |
| 49 | Th4 | 2 | 1996 | 0.25 µg/mL | - |
| 50 | FXb7 | 2 | 2000 | 0.25 µg/mL | - |
| 51 | Tb1 | 2 | 1996 | 0.25 µg/mL | - |
| 52 | Yb2 | 2 | 2000 | 0.25 µg/mL | - |
| 53 | Yb3 | 2 | 2001 | 0.25 µg/mL | - |
| 54 | NJ-3 | 2 | 2008 | 0.25 µg/mL | - |
| 55 | SC1 | 2 | 2008 | 0.25 µg/mL | - |
| 56 | YXL1 | 10 | 2008 | 0.25 µg/mL | - |
| 57 | WS1210 | 1 | 2013 | 0.25 µg/mL | - |
| 58 | HXb11 | 10 | 2001 | 0.25 µg/mL | - |
| 59 | WJ1 | 1 | 1998 | 0.25 µg/mL | - |
| 60 | CZL1 | ND | 1999 | 0.25 µg/mL | - |
| 61 | WZ | ND | 2006 | 0.25 µg/mL | - |
| 62 | HGb2 | ND | 2010 | 0.25 µg/mL | - |
| 63 | WXb8 | 2 | 2002 | 0.25 µg/mL | - |
| 64 | CZb3 | ND | 1999 | 0.25 µg/mL | - |
| 65 | XZ | ND | 2003 | 0.25 µg/mL | - |
| 66 | CH3 | 1 | 2001 | 0.125 µg/mL | - |
| 67 | Jb2 | 1 | 1996 | 0.125 µg/mL | - |
| 68 | Jb3 | 1 | 1996 | 0.125 µg/mL | - |
| 69 | JS6 | 1 | 1996 | 0.125 µg/mL | - |
| 70 | WJ4 | 1 | 2000 | 0.125 µg/mL | - |
| 71 | NJ-2 | 1 | 2008 | 0.125 µg/mL | - |
| 72 | CQ1 | 1 | 2009 | 0.125 µg/mL | - |
| 73 | PC2 | 2 | 1996 | 0.125 µg/mL | - |
| 74 | TD | 2 | 1996 | 0.125 µg/mL | - |
| 75 | JY-2 | 2 | 2008 | 0.125 µg/mL | - |
| 76 | HXL10 | 2 | 2001 | 0.125 µg/mL | - |
| 77 | YXb11 | 10 | 2001 | 0.125 µg/mL | - |
| 78 | YXb1 | 10 | 2008 | 0.125 µg/mL | - |
| 79 | CZb2 | ND | 1999 | 0.125 µg/mL | - |
| 80 | YYC | ND | 2005 | 0.125 µg/mL | - |
