# Supplementary material for: ErmF and ereD Are Responsible for Erythromycin Resistance in Riemerella anatipestifer
Source: PLoS One. 2015 Jun 24;10(6):e0131078. doi: 10.1371/journal.pone.0131078 (PMC4481100; doi:10.1371/journal.pone.0131078)
Supplement: S2 Table — (DOC) [file pone.0131078.s005.doc]

**Table S2. Primers used in this study**

| **Primers** | **Nucleotide sequence (5' to 3')** | **Product size** |
| --- | --- | --- |
| Primers used to detect *ermF* and *ereD* genes in *R. anatipestifer* | | |
| ermF P1 | 5′- GCCCGAAATGTTCAAGTTGT -3′ | 460 bp |
| ermF P2 | 5′- TTTCCGAAATTGACCTGACC -3′ |
| ereD P1 | 5′- CACCTTGGCATTTGAGTTTGGT -3′ | 713 bp |
| ereD P2 | 5′- TCATCGAAAGTCTTTGCCCCA -3′ |
| Primers used to detect other *erm, mphR* and *msrA* genes in *R. anatipestifer* | | |
| ermA P1 | 5′- ATCGGATCAGGAAAAGGACA -3′ | 537bp |
| ermA P2 | 5′- AGCCTGTCGGAATTGGTTTT -3′ |
| ermB P1 | 5′- AAGGGCATTTAACGACGAAACT -3′ | 543bp |
| ermB P2 | 5′- TTACTTTGGCGTGTTTCATTGC -3′ |
| ermC P1 | 5′- TGAAATCGGCTCAGGAAAAG -3′ | 564bp |
| ermC P2 | 5′- TCGTCAATTCCTGCATGTTT -3′ |
| ermD P1 | 5′- ATTTTTCCGGACAGCATTTG -3′ | 520bp |
| ermD P2 | 5′- ATTCTGACCATTGCCGAGTC -3′ |
| ermE P1 | 5′- CTGTTCGAGTGGGAGTTCGT -3′ | 325bp |
| ermE P2 | 5′- TCGGTTCGTTCTTCTGATCC -3′ |
| ermG P1 | 5′- AGGTGCAGGGAAAGGTCATT -3′ | 406bp |
| ermG P2 | 5′- TGCGCTATCCACTTTAGGTTT -3′ |
| ermT P1 | 5′- AACCGCCATTGAAATAGACC -3′ | 480bp |
| ermT P2 | 5′- GCTTGATAAAATTGGTTTTTGGA -3′ |
| ermX P1 | 5′- TCCATCATCGACCTTGTGAA -3′ | 620bp |
| ermX P2 | 5′- CGCAACCATGATTGTGTTTC -3′ |
| mphA P1 | 5′- GCCGATACCTCCCAACTGTA -3′ | 735bp |
| mphA P2 | 5′- ATACGTGAGGAGGAGCTTCG -3′ |
| mphR P1 | 5′- AATTTTTGCAGGTGCTCGTT -3′ | 318bp |
| mphR P2 | 5′- GGAGTTGGAAATCGTCGTGT -3′ |
| msrA P1 | 5′- AAACGGATAGAGCGTTTGGA -3′ | 488bp |
| msrA P2 | 5′- TCCGCCAACAATACCTTAGC -3′ |
| Primers used to distinguish *ermF* and *ermFU* | | |
| ermFU P1 | 5′- ACGCAGGCAGGGATTTT -3′ | 703 bp |
| ermFU P2 | 5′- CAGGACCTACCTCATAGACAAGT -3′ |
| ermF P1' | 5′- TTATCTCCCTGTAAACAGTGCTT -3′ | 816 bp |
| ermFU P2 | 5′- CAGGACCTACCTCATAGACAAGT -3′ |
| Primers used to amplify the *ermF*, *ermFU* and *ereD* cassettes for transfer | | |
| HXb2 ermFU P1 | 5′- TAACTAGTAATGGTGGGGCTTTGCGAAGA -3′ | 1620 bp |
| HXb2 ermFU P2 | 5′- TAGCATGCGTTATGCGGCAGCTTAAA -3′ |
| YXb15 ermFU P1 | 5′-GCTACTAGTTTTTCAAATTCAATTCTTCCCTATTTT -3′ | 1370bp |
| YXb15 ermFU P2 | 5′-TAGCATGCAAAAATCATCTTGACAACCAC -3′ |
| NJ4 ermFU P1 | 5′-CAACTAGTACATACGGCTCATAGATTCCATAAATTA -3′ | 1350bp |
| NJ4 ermFU P2 | 5′-ATGCATGCTACGAAGGATGAAATTTTTC -3′ |
| YZ-1 ermF P1 | 5′- GTACTAGTGATTAAGGACAATGGAACCTCCCAG -3′ | 1470 bP |
| YZ-1 ermF P2 | 5′- TAGCATGCAAAAATCATCTTGACAACCAC -3′ |
| SX ereD P1 | 5′- GCGACTAGTATTCAAATAATTCACATTTTATGTATTTCA -3′ | 1430 bp |
| SX ereD P2 | 5′- TAGCATGCAAAGAATAAAACGGCACCTC -3′ |
| Primers used to measure *ermF/ermFU* and *ereD* mRNA by real-time PCR | | |
| ermF/ermFU-r P1 | 5′- TTCGAAGTGGTGTCAAATATTCCTT -3′ | 100 bp |
| ermF/ermFU-r P2 | 5′- GGACAATGGAACCTCCCAGAA -3′ |
| ereD-r P1 | 5′- CAGTATATGGCAGAAAGTATTGATTGGT -3′ | 101 bp |
| ereD-r P2 | 5′- TCGACCGGAGTTTTTTGAATG -3′ |
| tbdR1-r P1 | 5′- AGCCGTTCTGGAGCTCGTT -3′ | 80 bp |
| tbdR1-r P2 | 5′- AATGTAACCCCCATTTTTTGAACA -3′ |
| Primers used to investigate the genetic environment of the *ermF* gene of strain HXb2 | | |
| ErmF-R1 SP1 | 5′- TGCAGTTCCGAAATTTCCTTTCA -3′ | 1^st^ walking |
| ErmF-R1 SP2 | 5′- CCAACTGTCAAATCAGCCCTGTTA -3′ |
| ErmF-L1 SP1 | 5′- TTTCCGAAATTGACCTGACCTGA -3′ |
| ErmF-L1 SP2 | 5′- AAGGACAATGGAACCTCCCAGAA -3′ |
| ErmF-w2 SP1 | 5′- TGTTTGGTTCGGTTTTACTATGTCG -3′ | 2^nd^ walking |
| ErmF-w2 SP2 | 5′- TGTTTATCAGTCATTTATGTGTTTTTCTATTC -3′ |
| ErmF-w2 SP3 | 5′- ATTTTGTCGTCTGTTCCGTGGA -3′ |
| ErmF-w3 SP1 | 5′- TCAATTCAGCACAACCGAAGCG -3′ | 3^rd^ walking |
| ErmF-w3 SP2 | 5′- TTTTCAACCCtGCACTTACCCC -3′ |
| ErmF-w3 SP3 | 5′- TGTCCACCTTGCAACAGTTCTG -3′ |
| Erm-w4 SP1 | 5′- TTGAGGTTCTTCCCTTTTGGA -3′ | 4^th^ walking |
| Erm-w4 SP2 | 5′- CGGAAAAAGTATTTGCGAAGG -3′ |
| Erm-w4 SP3 | 5′- ACCAACCGTAGCAGATGCTTT -3′ |
